# Predictive modeling of flavonoid efficacy against esophageal carcinoma: a comprehensive approach

**DOI:** 10.1038/s41598-025-23689-2

**Published:** 2025-11-12

**Authors:** Parham Pishro, Jalal A. Nasiri, Zahra Nasiri Sarvi, Sara Saeidi, Fatemeh B. Rassouli

**Affiliations:** 1 Department of Computer Science, Faculty of Mathematical Sciences, Ferdowsi University of Mashhad, Mashhad, Iran; 2 Department of Biology, Faculty of Science, Ferdowsi University of Mashhad, Mashhad, Iran; 3 Novel Diagnostics and Therapeutics Research Group, Institute of Biotechnology, Ferdowsi University of Mashhad, Mashhad, Iran

**Keywords:** Natural flavonoids, Esophageal carcinoma, Machine learning, Anticancer activity, Cancer, Drug discovery

## Abstract

Esophageal carcinoma poses a significant health challenge, particularly due to its notably high prevalence in East Asia, which underscores the urgent need for innovative treatment strategies. Natural flavonoids are polyphenolic compounds with significant potential to interact with cancer cell pathways; however, their therapeutic application remains constrained by the lack of comprehensive data and consistent experimental evidence. The current study aims to present a comprehensive framework for evaluating the anticancer potential of natural flavonoids, including seven flavones—luteolin, apigenin, chrysin, acacetin, cirsiliol, baicalein and eupatilin—and six flavonols—kaempferol, quercetin, galangin, myricetin, casticin, and gossyptin—against human esophageal carcinoma cells. Extensive experimental data from twenty-two research studies focusing on natural flavonoids and esophageal carcinoma cells were extracted based on specific inclusion criteria that emphasized dose, time, and cell viability. Seven machine learning models—K-Nearest Neighbors, Support Vector Machine, Logistic Regression, Decision Tree (DT), Random Forest, AdaBoost and XGBoost—were employed to predict the optimal dose and time required for flavonoids to achieve 50% cancer cell viability. Hyperparameters were fine-tuned for each algorithm, followed by 5-fold cross-validation on the train dataset to identify the most accurate predictive model. The performance of each algorithm was validated through rigorous hyperparameter optimization and validation on an independent test dataset. The DT model was identified as the most accurate predictor, leading to the development of a simplified model with low complexity that achieved an accuracy of 87.38%. This reliable model enables prediction of effective dosing parameters across diverse esophageal carcinoma cell lines. In conclusion, present study offers a robust yet accessible predictive tool for optimizing treatment strategies against esophageal carcinoma. By combining machine learning with natural compound research, this work exemplifies a transformative approach in oncology, accelerating the development of effective cancer therapies.

**Supplementary Information:**

The online version contains supplementary material available at 10.1038/s41598-025-23689-2.

## Introduction

Cancer is currently the second leading cause of death worldwide, and projections suggest a substantial increase in its impact over the next two decades. This translates to a lifetime risk of developing cancer for about one in five individuals, with mortality rates affecting approximately one in nine men and one in twelve women^1^. Esophageal cancer is a formidable neoplasm, ranking among the top causes of cancer-related mortality worldwide. The global epidemiology of esophageal cancer reveals pronounced geographic disparities, with the highest incidence rates observed in regions along the Esophageal Cancer Belt, spanning across Asia, including parts of northern China, southern Russia, northeastern Iran, Kazakhstan, and other Central Asian countries such as Turkmenistan, Mongolia, and Tajikistan^2–4^. Dietary factors, including the consumption of hot beverages, alcohol, and tobacco, are identified as significant risk factors contributing to the incidence of this cancer. The absence of clear early-stage symptoms significantly hinders early detection, resulting in only about 20% of cases being identified early. Consequently, patients diagnosed at later stages face a substantially lower 5-year survival rate^5–7^. The treatment of esophageal cancer employs a comprehensive approach, often combining multiple modalities to optimize outcomes. For early-stage cancers, esophagectomy is commonly performed, while chemical drugs and ionizing radiation are frequently utilized either preoperatively to reduce tumor size and improve surgical feasibility or postoperatively to prevent recurrence. Neoadjuvant chemoradiation therapy, which typically involves a combination of chemotherapy agents like cisplatin and 5-fluorouracil with radiation, is a standard strategy for advanced cases, aiming to enhance pathological response rates and improve overall survival^8–11^.

While conventional chemotherapy remains a therapeutic mainstay, its main limitation—systemic toxicity—is increasingly recognized as barriers to improving clinical outcomes. This leads to severe side effects such as fatigue, immune suppression, neuropathy, gastrointestinal toxicity and multidrug resistance, which renders therapies ineffective in a substantial proportion of advanced cancers^12–15^. In light of these challenges, there is a growing interest in alternative and complementary cancer treatments aimed at minimizing adverse effects while maintaining or enhancing therapeutic efficacy. Recent research has highlighted the potential of flavonoids isolated from medicinal plants, which have shown promising results in both preclinical and clinical cancer studies^16^. Flavonoids are poly-phenolic phytochemicals that are naturally present in various plant foods such as fruits, vegetables, herbs, olive oil, cacao, or nuts among others. To date, more than 10,000 different flavonoid compounds have been isolated and identified. These agents have been reported to be associated with a wide range of pharmaceutical effects, including anti-inflammatory, anticoagulant, antioxidative, immunomodulatory, antibacterial, antifungal, antiviral, and antihyperglycemic activities. Structurally, flavonoids share a basic C6-C3-C6 carbon backbone, consisting of two aromatic rings connected by a three-carbon bridge. Based on differences in substitutions on the benzene rings, flavonoids are categorized into seven subclasses, including flavones, flavanones, flavonols, flavan-3-ols, isoflavones, anthocyanidins, and chalcones^16^. For example, flavones include luteolin, apigenin, chrysin, acacetin, cirsiliol, baicalein, and eupatilin, whereas kaempferol, quercetin, galangin, myricetin, casticin, and gossyptin belong to the flavonol subclass. These compounds are commonly found in leaves, flowers, and fruits such as celery, parsley, red peppers, chamomile, mint, and Ginkgo biloba^17^. Importantly, flavonoids generally exhibit lower toxicity and fewer side effects compared to conventional cancer therapies^18–26^. Therefore, incorporating flavonoids into cancer treatment regimens may enhance patient tolerance and adherence, potentially leading to improved clinical outcomes.

Despite the encouraging potential of flavonoids in the treatment of gastrointestinal cancers^27^, there remains a lack of established protocols or formal recognition of flavonoid therapy as a legitimate treatment method. The variability in experimental and animal study outcomes highlights the need for further research to consolidate existing findings and clarify the role of flavonoids in cancer therapy. While clinical trials have explored escalating flavonoid doses, findings remain inconclusive and sometimes contradictory^28,29^.

Addressing these challenges, the present study introduces a novel computational framework designed to quantitatively evaluate the anticancer efficacy of diverse natural flavonoids specifically against esophageal carcinoma. Through systematic benchmarking and optimization of classical machine learning models, we not only rigorously assessed predictive performance but also developed a simplified and interpretable model that effectively translates complex biological data into actionable dosing strategies. This innovative methodological approach bridges the gap between experimental findings and practical therapeutic application, overcoming key limitations inherent in traditional studies. Moreover, by harnessing machine learning, our work lays the groundwork for accelerating flavonoid research, reducing experimental costs and time, and ultimately advancing their potential as viable adjuncts or alternatives in cancer therapy.

## Methods

### Data collection

**Fig. 1:**
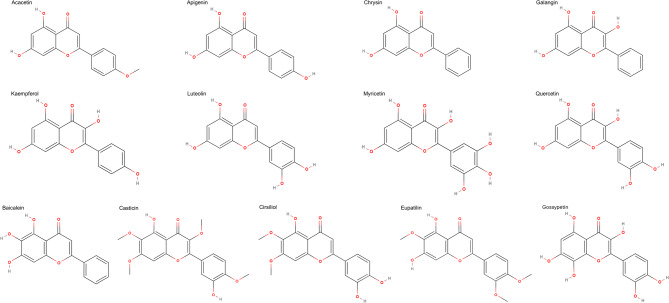
Chemical structures of natural flavonoids included in this study.

For data collection, a systematic review of in vitro studies involving human esophageal carcinoma cells was conducted, yielding essential quantitative data. A comprehensive search across multiple databases, including PubMed, Scopus and Google Scholar, identified relevant studies on the anticancer effects of natural flavonoids. Following a thorough screening of article abstracts to determine eligibility, three independent reviewers assessed the full texts of selected articles. Any reasons for excluding specific articles were meticulously documented, and disagreements were resolved through discussion until consensus was reached. In total, viability data for esophageal carcinoma cells treated with seven flavones—Luteolin, Apigenin, Chrysin, Acacetin, Cirsiliol, Baicalein, and Eupatilin—and six flavonols—Kaempferol, Quercetin, Galangin, Myricetin, Casticin, and Gossyptin (Fig. 1)—were extracted and included in the analysis^30–51^. Since the data were obtained from previously published studies, all original experiments involving esophageal carcinoma cell lines were conducted by the respective researchers under their appropriate ethical approvals.

The extracted data from the selected studies included various metrics such as cell viability percentages measured at different concentrations and time points across multiple cell lines. For both treatment and control groups, outcome measures comprised the number of replicates, means, and standard deviations (SD). To ensure precision and consistency, studies lacking this essential information in either tabular or graphical form were excluded. Furthermore, concentrations reported in units other than micromolar (µM) were converted for standardization. Data presented graphically were digitized using WebPlotDigitizer to enable comprehensive quantitative analysis. Table 1 summarizes the key findings from all the included studies.

**Table 1:** Characteristics of the included studies.

Studies	Flavonoids	Cell lines	Dose (µM)	Time (h)	Count
Chen et al. 2017^30^	Luteolin	KYSE 30	10–80	48	16
		KYSE 450		72	
Chen et al. 2021^31^	Chrysin	KYSE 30	1–50	72	24
		KYSE 150			
		KYSE 410			
		KYSE 450			
		YES 2			
Guo et al. 2022^32^	Baicalein	KYSE 150	6.7–20	24	4
				48	
Jia et al. 2021^33^	Cirsiliol	KYSE 140	5–20	24	18
				48	
		KYSE 450		72	
Li et al. 2023^34^	Quercetin	KYSE 30	50–200	48	8
		KYSE 150			
Qiao et al. 2019^35^	Casticin	TE 1	1–40	24	18
				48	
				72	
Qin et al. 2021^36^	Luteolin	TE 1	10–160	24	24
				48	
				72	
Qiu et al. 2019^37^	Apigenin	KYSE 30	3–300	48	5
Ren et al. 2016^38^	Galangin	TE 1	2.5–50	48	7
Wang et al. 2011a^39^	Baicalein	KYSE 510	10–80	24	46
	Chrysin				
	Galangin			48	
	Kaempferol			72	
Wang et al. 2011b^40^	Baicalein	OE 33	10–80	24	43
	Chrysin				
	Galangin			48	
	Kaempferol			72	
Wang et al. 2018^41^	Eupatilin	TE 1	2.5–50	24	30
				48	
				72	
				96	
Wang & Renquan 2023^42^	Acacetin	TE 1	20–60	24	18
				48	
		TE 10		72	
Wang & Zheng 2016^43^	Apigenin	OE 33	10–100	24	15
				48	
				72	
Xie et al. 2019^44^	Gossypetin	KYSE 30	20–60	48	9
		KYSE 450			
		KYSE 510			
Yao et al. 2016^45^	Kaempferol	KYSE 150	15–60	24	8
				48	
				72	
Zang et al. 2014^46^	Myricetin	KYSE 30	20–40	24	6
				48	
				72	
Zhang & Zhao 2008^47^	Apigenin	KYSE 510	10–80	24	137
	Chrysin				
	Kaempferol				
	Luteolin	OE 33			
	Myricetin			48	
	Quercetin			72	
Zhang et al. 2008^48^	Apigenin	OE 33	10–80	24	71
	Chrysin				
	Kaempferol				
	Luteolin				
	Myricetin			48	
	Quercetin			72	
Zhang et al. 2009^49^	Apigenin	KYSE 510	10–80	24	67
	Chrysin				
	Kaempferol				
	Luteolin				
	Myricetin			48	
	Quercetin			72	
Zhang et al. 2021^50^	Galangin	OE 19	10–80	48	8
				72	
Zhu et al. 2016^51^	Apigenin	KYSE 150	20–100	24	5

For the quantitative assessment of inter-study variability, six flavonoids—Apigenin, Quercetin, Luteolin, Kaempferol, Myricetin, and Chrysin—were selected due to their recurring evaluation across different studies employing identical cell lines, dosages, and exposure times, allowing for meaningful comparison. The Coefficient of Variation (CV) of viability measurements was calculated to characterize consistency and variability across these datasets. The CV values were consistently < 1, indicating relatively low variability and a high degree of homogeneity among the reported results (Table 2).

**Table 2:** CV values for studies using the same flavonoid against the same esophageal carcinoma cell line at comparable time points.

Studies	Flavonoids (40 µM)	Cell lines	Time (h)	CV
Wang et al. 2011a^9^	Chrysin	KYSE 510	24	0.116
Zhang & Zhao 2008^47^			48	0.206
Zhang et al. 2009^49^			72	0.270
Wang et al. 2011b^40^		OE 33	24	0.067
Zhang & Zhao 2008^47^			48	0.186
Zhang et al. 2008^48^			72	0.005
Wang et al. 2011a	Kaempferol	KYSE 510	24	0.015
Zhang & Zhao 2008^47^			48	0.197
Zhang et al. 2009^49^			72	0.596
Wang et al. 2011b^40^		OE 33	24	0.060
Zhang & Zhao 2008^47^			48	0.096
Zhang et al. 2008^48^			72	0.110
Zhang & Zhao 2008^47^	Apigenin	KYSE 510	24	0.004
			48	0.006
Zhang et al. 2009^49^			72	0.018
Zhang & Zhao 2008^47^		OE 33	24	0.011
			48	0.008
Zhang et al. 2008^48^			72	0.004
Zhang & Zhao 2008^47^	Luteolin	KYSE 510	24	0.013
			48	0.002
Zhang et al. 2009^49^			72	0.268
Zhang & Zhao 2008^47^		OE 33	24	0.002
			48	0.013
Zhang et al. 2008^48^			72	0.007
Zhang & Zhao 2008^47^	Myricetin	KYSE 510	24	0.000
			48	0.006
Zhang et al. 2009^49^			72	0.002
Zhang & Zhao 2008^47^		OE 33	24	0.002
			48	0.001
Zhang et al. 2008^48^			72	0.005
Zhang & Zhao 2008^47^	Quercetin	KYSE 510	24	0.010
			48	0.020
Zhang et al. 2009^49^			72	0.014
Zhang & Zhao 2008^47^		OE 33	24	0.004
			48	0.008
Zhang et al. 2008^48^			72	0.008

### Data preprocessing

To ensure the generalizability of the models, the dataset was split into train and test sets based on compound-level data splitting. Specifically, data from eight flavonoid compounds (Acacetin, Apigenin, Chrysin, Galangin, Kaempferol, Luteolin, Myricetin, and Quercetin), comprising 484 data points, were used for training. Data from five flavonoid compounds (Baicalein, Casticin, Cirsiliol, Eupatilin, and Gossypetin), consisting of 103 data points, were reserved exclusively for testing.

**Fig. 2:**
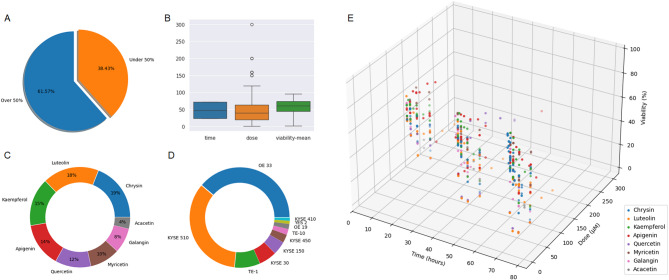
Percentage of viability in dataset (A). Box plot of time, dose and viability (B). Donut chart of flavonoids (C) and cell lines (D). The 3D scatter plot illustrating the relationship among time (hours), dose (µM) and percentage of viability (E).

Since the viability data were continuous, they were discretized to facilitate classification. Data points with viability values greater than 50% were labeled as 0 (indicating lower cytotoxicity), while those with viability values of 50% or below were labeled as 1 (indicating higher cytotoxicity). This cutoff corresponds to the widely accepted half-maximal inhibitory concentration (IC₅₀) threshold commonly used in cell biology, where values ≤ 50% indicate significant cytotoxic activity. As illustrated in Fig. 2-A, 61.57% of the data had viability values above 50%, and 38.43% were at or below this threshold. These binary labels were added as a new column to be used as the target variable in the modeling process.

Next, categorical features “Flavonoid” and “Cell Line” were encoded using one-hot encoding. Prior to encoding, the dataset contained 6 columns; following encoding, the total number of columns expanded to 22. This increase reflects the presence of 8 unique flavonoid types and 10 distinct cell lines in the dataset.

### Exploratory data analysis

Descriptive statistics for the features of time, dose, and viability were calculated and are summarized in Table 3. The distributions of these quantitative variables are visually represented through the box plot in Fig. 2-B. Additionally, donut charts illustrating the composition of flavonoid types and cell lines included in the dataset are shown in Fig .  2-C and -D, respectively.

**Table 3:** The descriptive statistics of time, dose and viability features.

	Count	Mean	STD	Min	25%	50%	75%	Max
Time	484	50.33	19.05	24	24	48	72	72
Dose	484	41.38	33.63	1	20	40	63.75	300
Viability	484	57.09	21.67	1.71	44.28	61.07	73.85	95.77

Figure 2-E presents a three-dimensional scatter plot depicting the relationships among time (hours) on the x-axis, dose (µM) on the y-axis, and viability (%) on the z-axis. Each data point is color-coded according to flavonoid type. This visualization highlights the diversity of the dataset and enables simultaneous examination of how exposure time and dose jointly affect cell viability. The plot reveals a general trend of decreased viability with higher doses and longer exposure times, while also suggesting potential clustering patterns unique to specific flavonoids. By integrating these three variables into one spatial representation, this 3D plot facilitates the identification of multi-variable interactions that may be less apparent in two-dimensional plots or correlation analyses.

### Modeling and evaluation

Following data preparation, a stratified 5-fold cross-validation was employed on the train set to ensure robust model evaluation. Stratification preserves the proportion of each class across all folds, thus providing balanced training and validation subsets. This approach facilitates reliable comparison among different models and hyperparameter configurations while minimizing the risk of biased performance estimates.

#### Algorithms

Seven machine learning algorithms were selected for model development based on their proven efficacy in cellular biology classification tasks:

##### K-Nearest neighbors (KNN)

A straightforward instance-based learning algorithm that classifies samples by majority vote of their closest neighbors in feature space. KNN is appreciated for its simplicity and computational efficiency; however, its performance depends heavily on the choice of the number of neighbors (*k*) and the distance metric.

##### Logistic regression (LR)

A probabilistic linear model widely used for binary classification. LR estimates the probability of class membership and is valued for its interpretability and ease of implementation. Nonetheless, it can struggle with multicollinearity among features or non-linear class boundaries.

$$p\left( x \right)=\frac{1}{{1+{e^{ - \left( {x - \mu } \right)/s}}}}$$

##### Support vector machine (SVM)

A powerful supervised learning algorithm that identifies the optimal hyperplane separating classes in a high-dimensional space. SVM handles high-dimensional data effectively and can model non-linear relationships via kernel functions. However, it may be computationally intensive, especially with large datasets or complex kernels.

$$\mathop {\hbox{min} }\limits_{{w,\xi }} \frac{1}{2}{\left\| w \right\|^2}+C\mathop \sum \limits_{{i=1}}^{n} {\xi _i}$$

$$s.t.{\text{~~~}}{y_i}\left( {{w^T}{x_i}+b} \right) \geqslant 1 - \xi {\text{~~~}},{\text{~~~}}\xi \geqslant 0$$

##### Decision tree (DT)

A hierarchical, rule-based classifier that partitions the data by selecting the most informative features at each node, forming a tree structure.

##### Random forest (RF)

An ensemble technique that constructs multiple DTs using random subsets of data and features. Aggregating predictions via majority voting, RF reduces overfitting and enhances model generalization compared to a single decision tree.

##### AdaBoost (AB)

An ensemble learning algorithm that builds a strong classifier by sequentially training a series of weak learners. At each iteration, the algorithm adjusts the weights of training samples—assigning greater weight to misclassified instances and reducing weight for correctly classified ones—thereby directing subsequent learners to focus on the more challenging examples. This adaptive weighting mechanism enhances the model’s overall performance by progressively improving accuracy at each step.

##### XGBoost (XGB)

An advanced gradient boosting framework that builds an ensemble of DTs in a stage-wise fashion. XGBoost incorporates regularization techniques to prevent overfitting and is optimized for computational efficiency and scalability, making it a robust and accurate choice for classification problems.

#### Performance metrics

The primary metric used for evaluating model performance was the accuracy score, defined as:

$${\text{Accuracy}}=\frac{{{\text{Number~of~Correct~Predictions}}}}{{{\text{Total~Number~of~Predictions}}}}$$

Additionally, a confusion matrix was generated for the final models to provide a detailed breakdown of true positives (TP), true negatives (TN), false positives (FP), and false negatives (FN).

$${\text{Accuracy}}=\frac{{TP+TN}}{{TP+TN+FP+FN}}$$

Hyperparameter tuning was conducted for all models to optimize their performance using the accuracy score as the evaluation criterion. The grid search method was employed, combined with cross-validation, to systematically explore the hyperparameter space. The number of neighbors $\:k$ for KNN, the kernel type for SVM, and the maximum depth of DTs were among the parameters fine-tuned.

### Importance of features

In order to evaluate the degree of relationship between quantitative features, the correlation matrix was computed using Pearson correlation coefficients, calculated as:

$${r_{xy}}=\frac{{\mathop \sum \nolimits_{{i=1}}^{n} \left( {{x_i} - \bar {x}} \right)\left( {{y_i} - \bar {y}} \right)}}{{\sqrt {\mathop \sum \nolimits_{{i=1}}^{n} {{\left( {{x_i} - \bar {x}} \right)}^2}} \sqrt {\mathop \sum \nolimits_{{i=1}}^{n} {{\left( {{y_i} - \bar {y}} \right)}^2}} }}$$

where ${x_i}$ and ${y_i}$​ represent individual feature values, and $\:\stackrel{-}{x}$ and $\:\stackrel{-}{y}$​ denote their respective means. This formula quantifies the linear relationship between two features, which includes very strong positive (0.70 to 1.00), strong positive (0.50 to 0.69), moderate positive (0.30 to 0.49), weak positive (0.01 to 0.29), no correlation (0.00), weak negative (-0.01 to -0.29), moderate negative (-0.30 to -0.49), strong negative (-0.50 to -0.69) and very strong negative (-0.70 to -1.00).

After calculating the correlation, the Exhaustive Feature Selection (EFS) method was applied to identify the most important features. This method systematically evaluates all possible feature subsets by iterating through all combinations and determines the optimal subset that maximizes model performance. DT was used as the evaluator, and the metric applied for evaluating feature subsets was the ${R^2}$ score, calculated as follows:

$${R^2}=1 - \frac{{\sum {{\left( {{y_i} - {{\hat {y}}_i}} \right)}^2}}}{{\sum {{\left( {{y_i} - \bar {y}} \right)}^2}}}$$

where ${y_i}$ represents the observed values, ${\hat {y}_i}$​ are the predicted values, and $\bar {y}$​ denotes the mean of the observed values. The ${R^2}$ score indicates the proportion of variance in the dependent variable that can be predicted from the independent variables, with values closer to 1 signifying better performance.

## Results

This section is organized into five subsections. The first subsection details the hyperparameter tuning process, where KNN, LR, SVM, DT, RF, AB, and XGB models were initially developed using the default settings outlined in section “Algorithms”. The objective of this step was to optimize model performance by identifying the best hyperparameters. The second subsection presents the outcomes of the hyperparameter tuning, accuracy of each model on the train set, and optimal dose and time for flavonoids on the train set. The third subsection focuses on the correlation analysis and the relative importance of each feature. The fourth subsection reports on the prediction performance of the seven machine learning models (KNN, LR, SVM, DT, RF, AB, and XGB). The results of these models with the best hyperparameters are compared. Finally, the fifth subsection introduces a simplified model derived from the best-performing algorithms, designed to define the optimal time and dosage needed to achieve 50% cell viability.

### Hyperparameter tuning

Hyperparameters are parameters set prior to training, and tuning them significantly influences model performance. Default settings were applied during initial data selection; however, tuning hyperparameters for each model is essential to improve performance. For categorical hyperparameters, accuracy scores were compared, while for numeric hyperparameters, accuracy scores were plotted across a range of values to identify the optimal settings. The results presented in this section were obtained using stratified 5-fold cross-validation on the train set only.

#### KNN

In optimizing the KNN model, three hyperparameters were tuned: number of neighbors (n_neighbors), weights, and metric. These parameters were adjusted to maximize the accuracy score. The performance for different combinations of weights and metrics is summarized in Table 4. As shown, the “distance” weight outperformed “uniform,” and the “euclidean” metric achieved higher accuracy compared to alternatives such as “manhattan.” Therefore, “distance” was selected as the weight and “euclidean” as the metric for further evaluation.

**Table 4:** The result of accuracy score with different weights and metric values.

Weights		Metric		
Uniform	Distance	Euclidean	Manhattan	Minkowski
0.7996	0.9112	0.9112	0.9071	0.9112

**Fig. 3:**
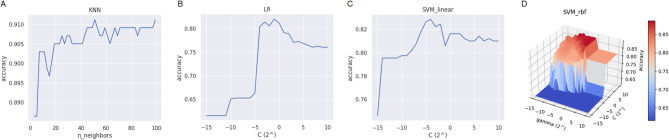
The result of accuracy score with different n_neighbors values of KNN (A). The result of accuracy score with different C values of LR from ${2^{ - 15}}$ to ${2^{10}}$ (B). The result of accuracy score with different C values for linear kernel of SVM from ${2^{ - 15}}$ to ${2^{10}}$ (C). The result of accuracy score with different combinations of C and $\gamma$ values for RBF kernel of SVM from ${2^{ - 15}}$ to ${2^{10}}$ (D).

For n_neighbors, 49 values ranging from 3 to 99 with a step size of 2 were tested. Accuracy scores for these values are plotted in Fig. 3-A, indicating the highest accuracy at n_neighbors = 51. The optimal hyperparameter settings for KNN are summarized as: weights = “distance,” metric = “euclidean,” and n_neighbors = 51.

#### LR

Two hyperparameters were optimized for LR. The first is the penalty, which specifies the type of normalization used in penalization. The second is the inverse of regularization strength (C), where smaller values correspond to stronger regularization. Initially, the model’s performance was evaluated with different penalty types, and as shown in Table 5, “L1” was selected for yielding the highest accuracy.

**Table 5:** The result of accuracy score with different penalty values.

Penalty		
None	L1	L2
0.7647	0.8203	0.7813

For C, 26 values between $\:{2}^{-15}$ to $\:{2}^{10}$ were set to C as shown in Fig. 3-B. The accuracy score reaches the highest at C = $\:{2}^{-1}$. The summary of the best settings for LR is: penalty = “L1” and C = $\:0.5$.

#### SVM

SVM were evaluated with three different kernels: linear, radial basis function (RBF), and polynomial, each having specific hyperparameters. The hyperparameter tuning process for each kernel is detailed below:

- **Linear Kernel**.

The linear kernel has one hyperparameter, C, which controls the trade-off between achieving a low error on the training data and minimizing model complexity. Values of C ranging from $\:{2}^{-15}$ to $\:{2}^{10}$ were tested, as shown in Fig. 3-C. The highest accuracy was observed around C = $\:{2}^{-4}$.

- **RBF Kernel**.

The RBF kernel includes two hyperparameters: C and $\:\gamma\:$. The second hyperparameter, $\:\gamma\:$, defines the influence of a single training example. They were tuned over the same range as the linear kernel $\:{2}^{-15}$ to $\:{2}^{10}$, and the results are presented in Fig. 3-D.

- **Polynomial Kernel**.

Despite extensive tuning, the polynomial kernel did not reach the accuracy levels achieved by the RBF kernel and demonstrated unstable predictions. Furthermore, it required considerably longer computation times compared to the other kernels. Consequently, the polynomial kernel was excluded from further analysis.

Among these kernels, the RBF kernel has the best accuracy score. The summary of the best settings for SVM is: kernel = “rbf”, C = $\:{2}^{10}$, and $\:\gamma\:$ = $\:{2}^{-6}$.

#### DT

**Fig. 4:**
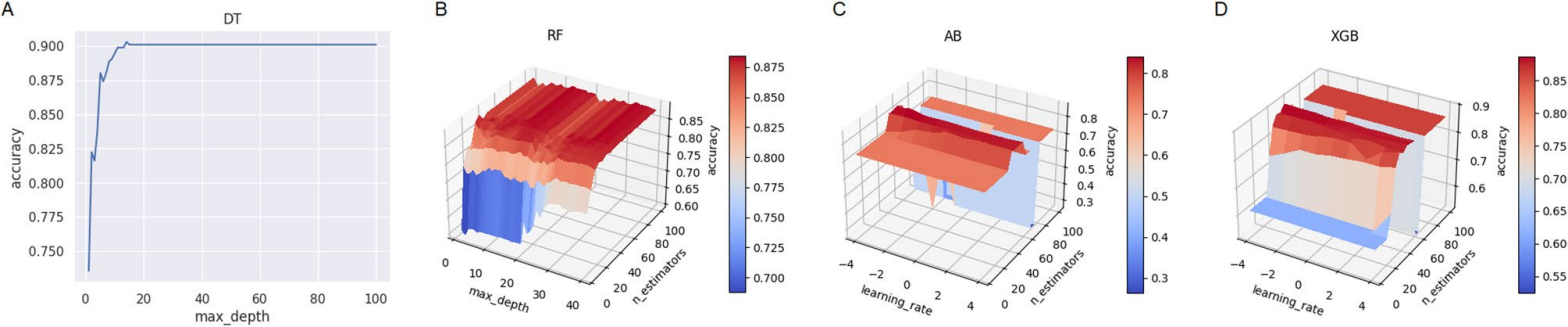
The result of accuracy score with different max_depth values of DT (A). The result of accuracy score with different combinations of max_depth of RF values from 1 to 40 and n_estimators values of RF from 5 to 100 (B). The result of accuracy score with different combinations of learning_rate of AB from ${10^{ - 4}}$ to ${10^4}$ and n_estimators values of AB from 5 to 100 (C). The result of accuracy score with different combinations of learning_rate of XGB from ${10^{ - 4}}$ to ${10^4}$ and n_estimators values of XGB from 5 to 100 (D).

For the optimization of DT model, five hyperparameters were adjusted to evaluate their impact on performance. These hyperparameters and their descriptions are provided in Table 6. The accuracy scores for different values of the criterion and splitter parameters are presented in Table 7. The “best” splitter option achieved higher accuracy compared to “random,” while the “gini” criterion outperformed “entropy.” The model’s performance across various tree depths is shown in Fig. 4-A. Performance stabilized at around a depth of 20, indicating that increasing the tree depth beyond this point does not improve accuracy and may lead to higher computational cost. The highest accuracy was observed at a max_depth of 14, which was therefore selected as the optimal value.

**Table 6:** Decision tree hyperparameters settings.

Hyperparameters	Description	Values
*criterion*	It measures the quality of a split	Gini and entropy
*splitter*	A strategy that is used to select the split at each node	Best and random
*max_depth*	The maximum depth of the tree	[1–100]
*minimum_samples_split*	The minimum number of samples required to split	[2–102], step = 10
*minimum_samples_leaf*	The minimum number of samples required to be a leaf node	[1–101], step = 10

**Table 7:** The result of accuracy score with different criterion and splitter values.

Criterion		Splitter	
Gini	Entropy	Random	Best
0.9030	0.8720	0.8906	0.9030

Regarding the hyperparameters minimum_samples_split and minimum_samples_leaf, both exhibit a similar trend: as their values increase, the accuracy generally decreases. This is expected because larger values require a greater portion of the data to perform a split, resulting in coarser partitioning. For both parameters, the best performance was achieved with the smallest values, which correspond to the defaults—1 for minimum_samples_leaf and 2 for minimum_samples_split. The optimized hyperparameter settings for DT are summarized as follows: criterion = “gini”, splitter = “best”, max_depth = 14, minimum_samples_split = 2, and minimum_samples_leaf = 1.

#### RF

Tuning RF is similar to DT, as RF is essentially an ensemble of multiple DTs. The hyperparameters max_depth, criterion, minimum_samples_leaf, and minimum_samples_split are shared between the two models. The additional hyperparameter specific to RF is the number of trees in the forest (n_estimators). The considered values for this hyperparameter are from 5 to 100 with step size of 5. The accuracy score reaches maximum value at 5 estimators. For *max_depth*, values ranging from 1 to 40 were evaluated, with the highest accuracy achieved at 11. The performance trends for n_estimators and max_depth are shown in Fig. 4-B.

The accuracy score of different criterions were 0.8906 and 0.8659 for gini and entropy, respectively, which indicated that ‘‘gini” value for criterion has better accuracy score than ‘‘entropy”. The other two hyperparameters are the same as DT. Both the minimum_samples_split and minimum_samples_leaf have the same pattern in which the accuracy score decreases as the value increases. Thus, the best value is the smallest value. The summary of the best settings for RF is: n_estimators = 5, criterion = gini, max_depth = 11, minimum_samples_split = 2, and minimum_samples_leaf = 1.

#### AB

AB model involves two key hyperparameters: learning_rate and the number of estimators (n_estimators). For the learning_rate, 9 values ranging from $\:{10}^{-4}$ to $\:{10}^{4}$ were tested. Additionally, for n_estimators, 20 values ranging from 5 to 100, with a step size of 5, were tested. The accuracy scores for different combinations of these hyperparameters are illustrated in Fig. 4-C. The optimal settings for AB were identified as learning_rate = 1 and n_estimators = 15.

#### XGB

Similarly, XGB model has two key hyperparameters: learning_rate and n_estimators, with ranges identical to those used for AB. The performance of different learning_rate and n_estimators values is illustrated in Fig. 4-D. The optimal settings for XGB were identified as learning_rate = 1 and n_estimators = 10.

### Results of hyperparameter tuning

**Fig. 5:**
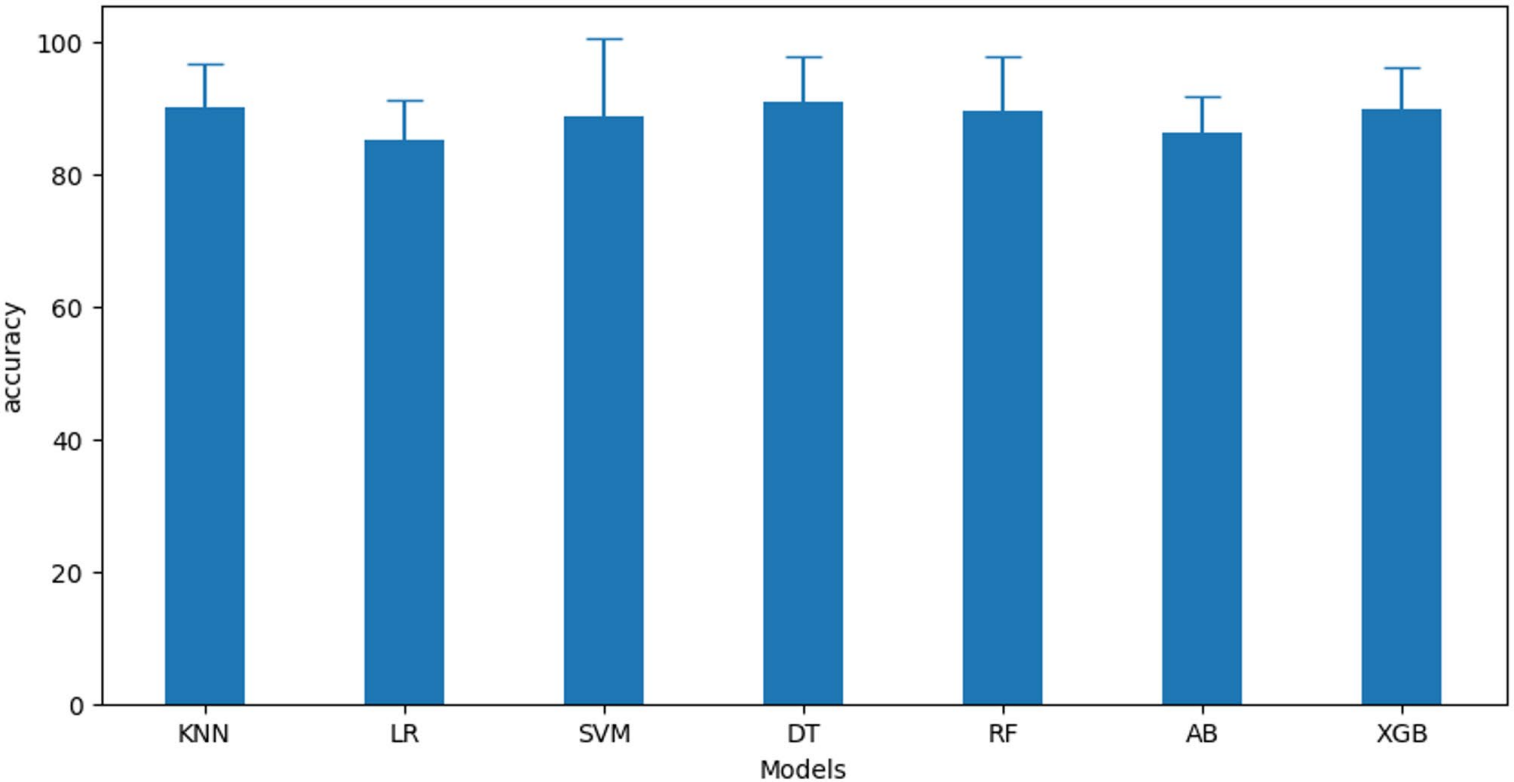
Accuracy score of each final model on the train set.

The best hyperparameters for each algorithm, summarized in Table 8, were used to build the final models. These models were evaluated using stratified 5-fold cross-validation on the train dataset, with the results shown in Fig. 5. Among the models, DT achieved the highest accuracy of 90.91% ± 6.88. The performance of the remaining models ranked as follows: KNN: 90.29% ± 6.53, XGB: 89.88% ± 6.49, RF: 89.68% ± 8.09, SVM: 88.86% ± 11.76, AB: 86.36% ± 5.40, and LR: 85.33% ± 5.92.

**Table 8:** Summary of the best setting for each model.

Models	Hyperparameters
KNN	(metric=’euclidean’, n_neighbors = 51, weights=’distance’)
LR	(C = 0.5, penalty=’l1’)
SVM	$\left( {{\text{C}}={2^{10}},{\text{~gamma}}={2^{ - 6}},{\text{~kernel}}={\text{'rbf'}}} \right)$
DT	(criterion=’gini’, max_depth = 14, min_samples_leaf = 1, min_samples_split = 2, splitter=’best’)
RF	(criterion=’gini’, max_depth = 11, min_samples_leaf = 1, min_samples_split = 2, n_estimators = 5)
AB	(learning_rate = 1, n_estimators = 15)
XGB	(learning_rate = 1, n_estimators = 10)

Based on the best-performing model, the optimal dose and time parameters for each flavonoid used in the train set are presented in Table 9. Since the test set includes different flavonoids than the train set, this feature could not be used for prediction. Moreover, future applications may involve different cell lines. Therefore, the optimal values obtained for training flavonoids and features of the test set only include dose and time, the importance of which is shown in the next subsection.

**Table 9:** Optimal dose and time for each training flavonoid.

Flavonoids	Optimal dose (µM) and time (h)
Acacetin	(dose > 30.0, time > 60) or (dose > 50.0)
Apigenin	(dose > 35.0, time > 36) or (dose > 77.5)
Chrysin	dose > 22.5, time > 36
Galangin	dose > 25.0, time > 36
Kaempferol	dose > 35.0, time > 36
Luteolin	dose > 35.0, time > 36
Myricetin	dose > 30.0, time > 36
Quercetin	(dose > 30.0, time > 36) or (dose > 60.0)

### Importance of features

**Fig. 6:**
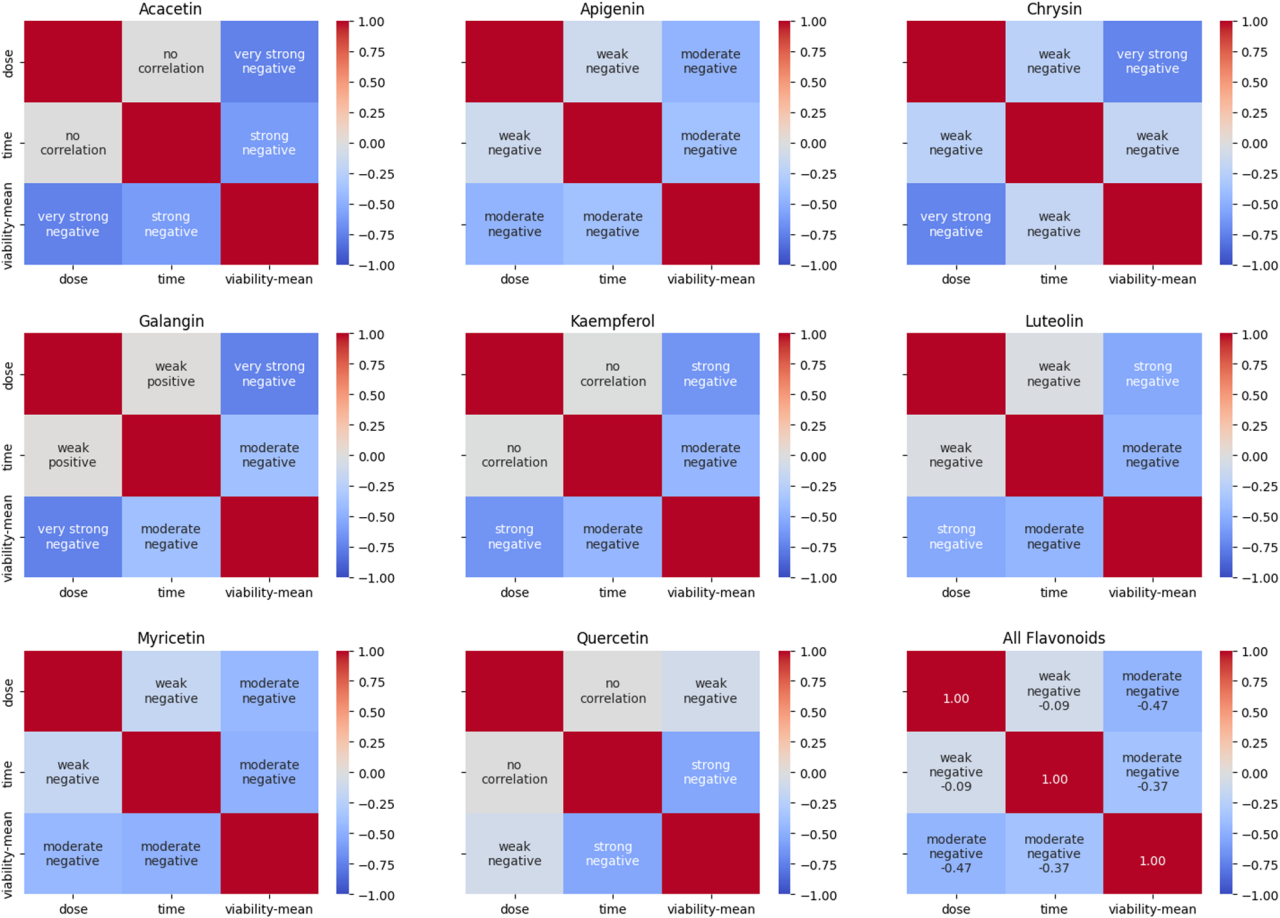
Heatmaps of correlations between dose, time and viability for each flavonoid.

The correlation heatmap for each flavonoid reveals a strong negative correlation between dose and viability, which is notably stronger than the correlation between time and viability. The average correlation coefficients across flavonoids are − 0.55 for dose–viability and − 0.43 for time–viability, while the weighted averages are − 0.54 and − 0.40, respectively. Both sets of averages confirm that dose has a stronger negative correlation with viability than time. These relationships are also reflected in the combined correlation heatmap of all flavonoids (Fig. 6).

**Fig. 7:**
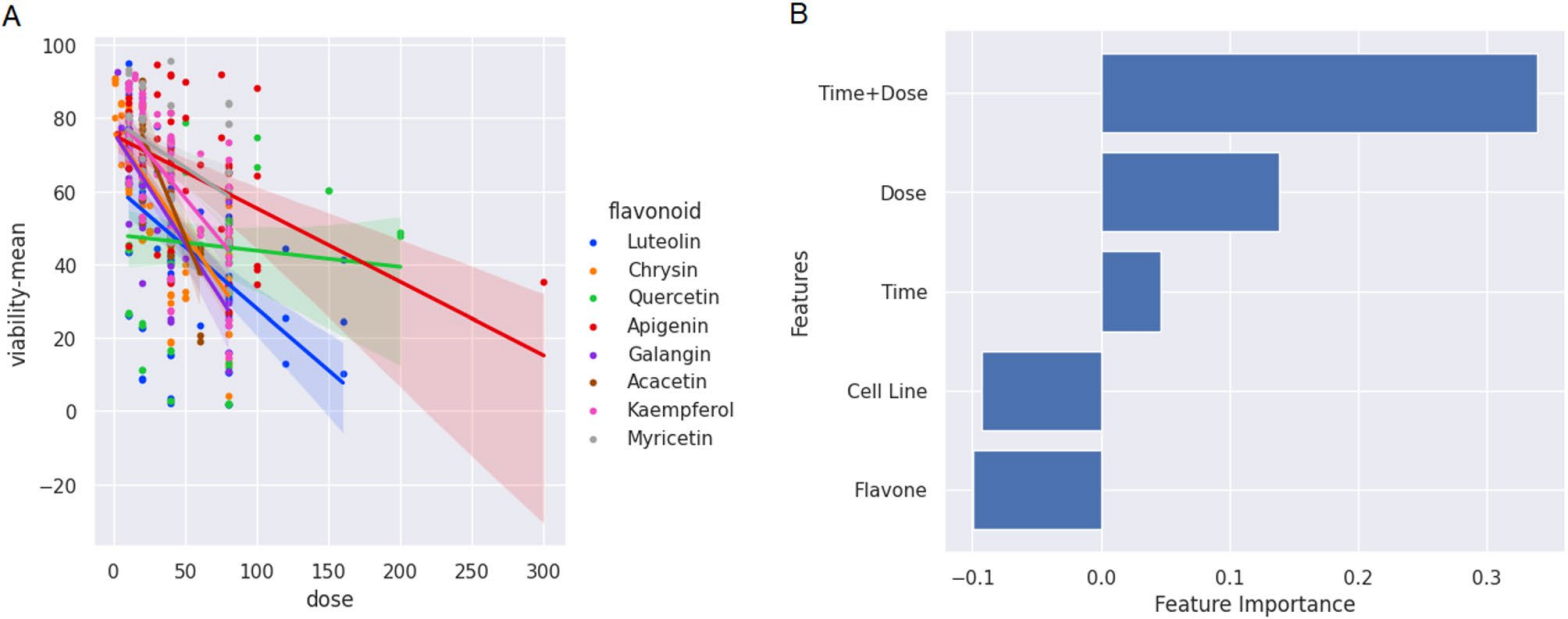
A 2D scatter plot illustrating the correlation between dose (µM) and viability (%) across various flavonoids. Each flavonoid is distinguished a different color in the plot (A). Importance of features using EFS (B).

Figure 7-A presents a 2D scatter plot illustrating the correlation between dose (the feature with the highest correlation) and viability across various flavonoids. Figure 7-B displays the importance of each feature. According to this figure, the cell line and flavonoid features contribute less to the prediction. A negative R² value in Python indicates that the model was unable to make meaningful predictions based on that feature (a value equivalent to $\:{r}^{2}=0$). The importance of time and dose surpasses that of other features, and their combination was identified as the most predictive by EFS method.

### Final model and evaluation

**Fig. 8:**
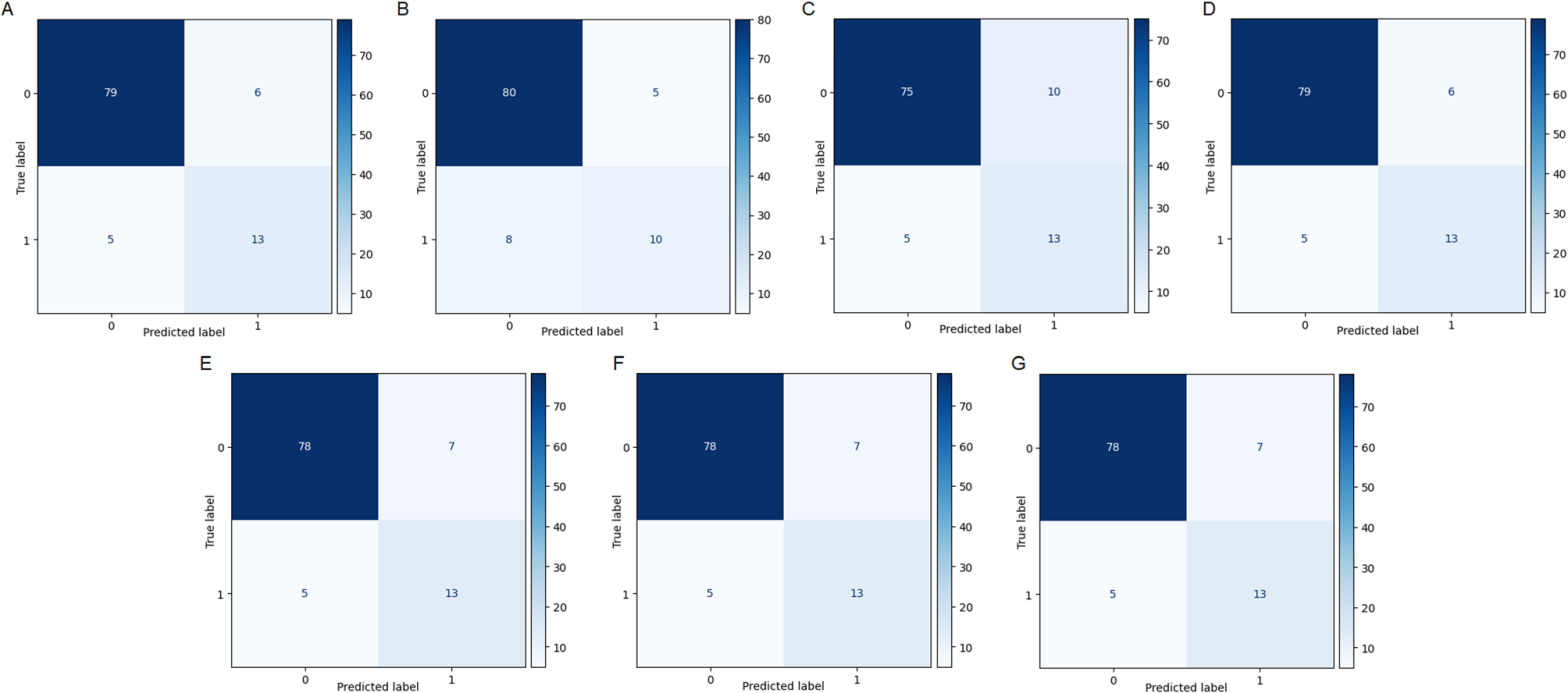
Confusion matrices of each model; KNN (A), LR (B), SVM (C), DT (D), RT (E), AB (F), and XGB (G).

The test dataset contains 103 samples: **85 with viability over 50% (class 0)** and **18 with viability under 50% (class 1)**. The confusion matrices for all models were plotted, as shown in Fig. 8-A-G. These matrices summarize the following:

- **True Positive (TP)**: Correctly classified data with above 50% viability.
- **True Negative (TN)**: Correctly classified data with below 50% viability.
- The overall performance of the models indicates that predictions are generally accurate, as the sum of TP and TN exceeds the sum of False Positive (FP) and False Negative (FN) in all cases. However, an imbalance in the dataset resulted in more TP than TN across all models, which is also reflected in the FP and FN distributions.

Given the imbalanced nature of the dataset, the “weighted average” (weighted avg) is a more appropriate metric for evaluation, as it accounts for class proportions while computing the evaluation metrics. Table 10 provides a summary of classification reports (such as precision, recall, F1-score, and accuracy) for all seven models. According to these metrics, the DT and KNN models consistently outperform the others, demonstrating superior prediction performance across all metrics. Due to excellent results of DT model in train and test sets, and also the interpretability of DT, this model was chosen as the primary predictive model for subsequent analyses.

**Table 10:** Results of precision, recall, F1-score, and accuracy for all seven models on the test set.

Metrics/models	KNN	LR	SVM	DT	RT	AB	XGB
Precision	0.90	0.87	0.87	0.90	0.89	0.89	0.89
Recall	0.89	0.87	0.85	0.89	0.88	0.88	0.88
f1-score	0.89	0.87	0.86	0.89	0.89	0.89	0.89
Accuracy	0.89	0.87	0.85	0.89	0.88	0.88	0.88

### The simple model

**Fig. 9:**
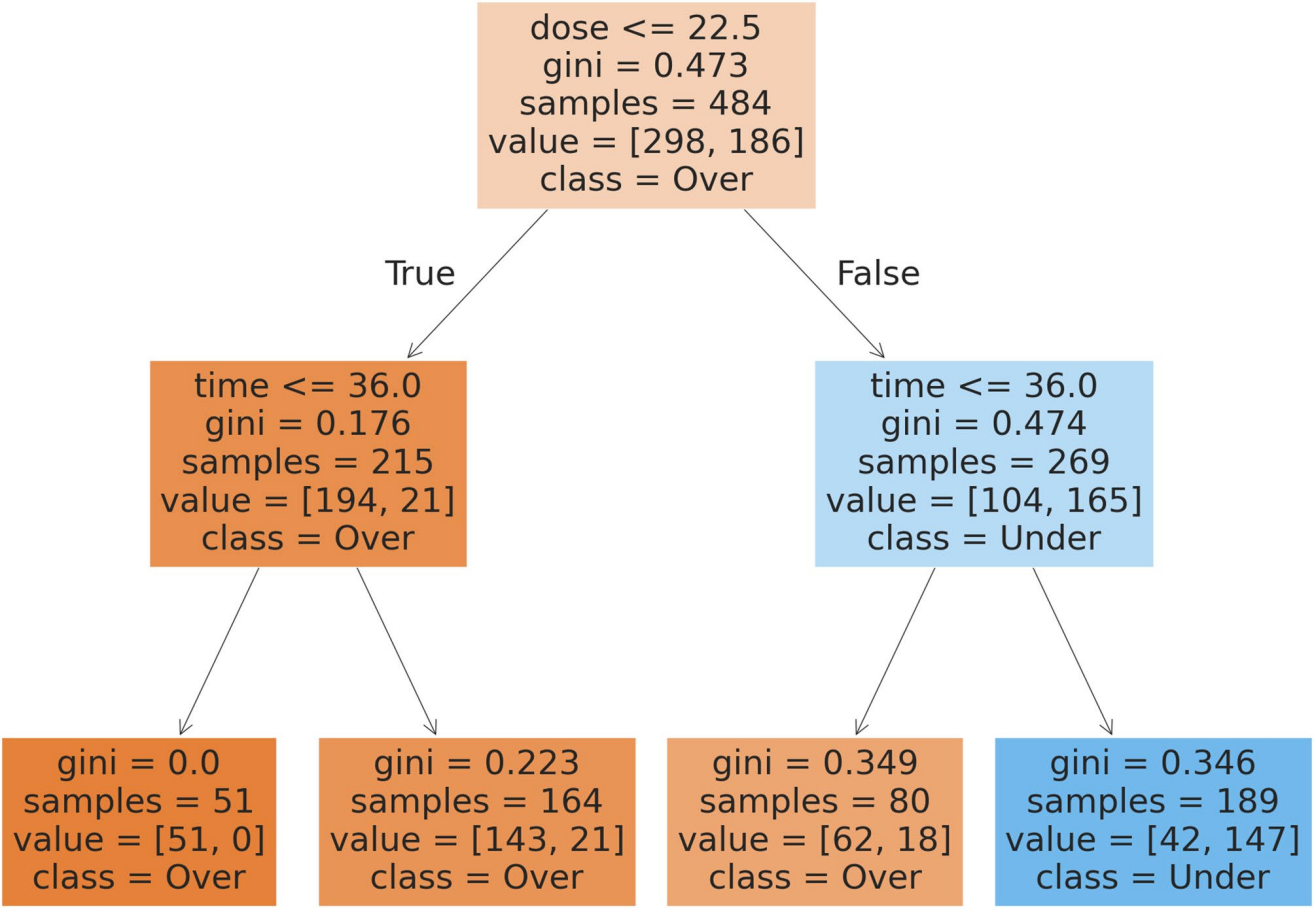
Diagram of the simple model.

After establishing DT as the best-performing model, a simplified model with reduced depth was developed, achieving an accuracy of 87.38%. This streamlined model enables identification of practical dose and time thresholds to guide researchers effectively. The classification report, including precision, recall, f1-score, and accuracy for the simplified model, is presented in Table 11. Furthermore, Fig. 9 illustrates that to achieve cell viability below 50%, the dose should be at least 22.5 µM (preferably ≥ 25 µM) and the exposure time should be a minimum of 36 h. Failure to meet these conditions substantially decreases the likelihood of reducing viability below 50%.

**Table 11:** Classification report for the simple model.

Simple model	Precision	Recall	f1-score	Support
0	0.94	0.91	0.92	85
1	0.62	0.72	0.67	18
Accuracy			0.87	103
Macro avg	0.78	0.81	0.79	103
Weighted avg	0.88	0.87	0.88	103

## Discussion

Esophageal cancer is a significant health concern, especially in East Asia, where countries such as China and Japan report some of the highest mortality rates^52,53^. Chemotherapy is widely recognized as the primary treatment for this neoplasm, but its use is often associated with severe side effects that demand close monitoring. To address this issue, there is increasing interest in exploring alternative or complementary treatments that aim to reduce toxicity while preserving therapeutic effectiveness. Among these approaches, flavonoids derived from medicinal plants have gained attention for their potential anticancer properties. Both preclinical and clinical studies have demonstrated promising outcomes, suggesting that these natural compounds may offer a safer and effective option for cancer management^54,55^. By integrating such alternatives into existing protocols, it may be possible to enhance therapeutic outcomes while mitigating the debilitating side effects of conventional chemotherapy.

The integration of machine learning algorithms is transforming cancer research by facilitating the discovery of new therapeutic agents and enhancing treatment strategies. By leveraging extensive databases that catalog the structural and pharmacological characteristics of natural anticancer agents, machine learning algorithms can uncover intricate patterns and predict therapeutic effects with remarkable precision. For instance, the PECAN model employs deep learning techniques to assess cytostatic effects across various cancer cell lines. It excels in identifying structural features associated with anticancer potential and achieves high predictive accuracy^56^. Beyond analyzing complex datasets, machine learning models provide accurate target identification, optimize drug dosages, and refine administration schedules, which improves treatment effectiveness while reducing side effects. This approach circumvents traditional trial-and-error and resource-intensive empirical methods by facilitating comprehensive exploration of therapeutic parameters via computational simulations. As a result, promising therapeutic candidates can be identified more rapidly, significantly reducing the reliance on extensive preliminary testing.

The present machine learning-based study investigated the anticancer potential of natural flavonoids against esophageal cancer. It focused on evaluating the ability of these compounds to reduce the viability and proliferation of human esophageal cancer cells, using extensive in vitro data quantifying their cytotoxic effects. Seven machine learning models—KNN, SVM, LR, DT, RF, AB, and XGB—were employed to predict the optimal dose and exposure time for natural flavones and flavonols to achieve 50% cancer cell viability. Hyperparameters for each algorithm were fine-tuned, and their performance was validated on an independent test dataset. Upon identification of the DT algorithm as the most accurate predictor, we developed a simple model with low depth but an accuracy of 87.38%. Intriguingly, our simplified yet robust model enables precise predictions of effective dosing and timing parameters across various esophageal carcinoma cell lines. Our findings also revealed a remarkable consistency in the anticancer properties of the flavonoids studied, all demonstrating dose-response relationships potentially applicable across multiple cell lines. This predictive approach offers an efficient method for identifying treatment parameters while reducing the time and cost associated with conventional experimental research.

The present study encountered several limitations that warrant careful consideration in future research. A key constraint was the limited availability of high-quality experimental data on flavonoids, despite being widely studied natural compounds in oncology. This scarcity was partly due to the prevalent use of problematic or contaminated esophageal carcinoma cell lines—including EC1, EC9706, and EC109—as well as mixed cell line samples such as TE7 and TE13. Additionally, to maintain clinical relevance and dataset consistency, studies employing murine esophageal cell lines were excluded, focusing exclusively on human-derived data to ensure greater homogeneity. Another significant challenge was the inherent biological and methodological variability common to independent in vitro experiments, including differences in flavonoid dosage and treatment duration, which prevented defining inter-study variability across all the included studies. Such variability could potentially affect the robustness and predictive accuracy of the developed models.

Future investigations would benefit from broadening the data collection framework to encompass additional gastrointestinal malignancies, such as gastric and colorectal adenocarcinomas. This expansion would not only increase dataset size and homogeneity but also enhance the generalizability of predictive models, enabling more reliable and clinically meaningful prognostications.

In conclusion, this study presents a simplified yet robust predictive model achieving commendable accuracy in determining optimal dosing across various esophageal carcinoma cell lines. By integrating machine learning with natural compound research, our approach exemplifies a promising paradigm shift in oncology, facilitating the acceleration of effective cancer therapies.

## Supplementary Information

Below is the link to the electronic supplementary material.

Supplementary Material 1

## Data Availability

The curated dataset and accompanying Python scripts used in this study are publicly accessible to promote transparency and reproducibility. They can be found in the following repository: [https://github.com/ParhamPishro/Flavonoid](https:/github.com/ParhamPishro/Flavonoid).
